# Tanycytes and the Control of Thyrotropin-Releasing Hormone Flux Into Portal Capillaries

**DOI:** 10.3389/fendo.2019.00401

**Published:** 2019-06-25

**Authors:** Adair Rodríguez-Rodríguez, Iván Lazcano, Edith Sánchez-Jaramillo, Rosa María Uribe, Lorraine Jaimes-Hoy, Patricia Joseph-Bravo, Jean-Louis Charli

**Affiliations:** ^1^Departamento de Genética del Desarrollo y Fisiología Molecular, Instituto de Biotecnología, Universidad Nacional Autónoma de México, Cuernavaca, Mexico; ^2^Departamento de Neurobiología Celular y Molecular, Instituto de Neurobiología, Universidad Nacional Autónoma de México, Juriquilla, Mexico; ^3^Laboratorio de Neuroendocrinología Molecular, Dirección de Investigaciones en Neurociencias, Instituto Nacional de Psiquiatría Ramón de la Fuente Muñiz, Mexico City, Mexico

**Keywords:** thyrotropin releasing hormone (TRH), thyroid hormone, tanycyte, median eminence, thyrotropin (TSH–thyroid-stimulating hormone), TRH degrading ectoenzyme, paraventricular (PVN), third ventricle

## Abstract

Central and peripheral mechanisms that modulate energy intake, partition and expenditure determine energy homeostasis. Thyroid hormones (TH) regulate energy expenditure through the control of basal metabolic rate and thermogenesis; they also modulate food intake. TH concentrations are regulated by the hypothalamus-pituitary-thyroid (HPT) axis, and by transport and metabolism in blood and target tissues. In mammals, hypophysiotropic thyrotropin-releasing hormone (TRH) neurons of the paraventricular nucleus of the hypothalamus integrate energy-related information. They project to the external zone of the median eminence (ME), a brain circumventricular organ rich in neuron terminal varicosities and buttons, tanycytes, other glial cells and capillaries. These capillary vessels form a portal system that links the base of the hypothalamus with the anterior pituitary. Tanycytes of the medio-basal hypothalamus express a repertoire of proteins involved in transport, sensing, and metabolism of TH; among them is type 2 deiodinase, a source of 3,3′,5-triiodo-L-thyronine necessary for negative feedback on TRH neurons. Tanycytes subtypes are distinguished by position and phenotype. The end-feet of β2-tanycytes intermingle with TRH varicosities and terminals in the external layer of the ME and terminate close to the ME capillaries. Besides type 2 deiodinase, β2-tanycytes express the TRH-degrading ectoenzyme (TRH-DE); this enzyme likely controls the amount of TRH entering portal vessels. TRH-DE is rapidly upregulated by TH, contributing to TH negative feedback on HPT axis. Alterations in energy balance also regulate the expression and activity of TRH-DE in the ME, making β2-tanycytes a hub for energy-related regulation of HPT axis activity. β2-tanycytes also express TRH-R1, which mediates positive effects of TRH on TRH-DE activity and the size of β2-tanycyte end-feet contacts with the basal lamina adjacent to ME capillaries. These end-feet associations with ME capillaries, and TRH-DE activity, appear to coordinately control HPT axis activity. Thus, down-stream of neuronal control of TRH release by action potentials arrival in the external layer of the median eminence, imbricated intercellular processes may coordinate the flux of TRH into the portal capillaries. In conclusion, β2-tanycytes appear as a critical cellular element for the somatic and post-secretory control of TRH flux into portal vessels, and HPT axis regulation in mammals.

## Introduction

Thyroid Hormones (TH) are pleiotropic hormones that regulate body physiology throughout vertebrate life. TH are critical in the perinatal period; anomalies in maternal thyroid status have a severe impact on central nervous system development (1, 2). Growth and adult life are dependent on energy homeostasis, maintained by central and peripheral mechanisms modulating energy intake, partition, and expenditure. When organisms are challenged by novel environments, whether changing availability of nutrients, climate, reproductive, or internal energy demands, they use mechanisms of adaptation involving, among others, adjustments of TH levels (3). In coordination with sympathetic activation, TH play an important role in maintaining basal metabolic rate and thermogenesis in homeothermic organisms (4), they also control carbohydrate and lipid metabolism (3) and have a direct influence on hypothalamic nuclei that control energy intake and expenditure (5–8). Alterations in TH homeostasis are accompanied by several pathologies related to energy imbalances (9, 10).

The hypothalamus-pituitary-thyroid (HPT) axis of mammals integrates TH negative feedback, nutritional-, metabolic-, stress-related information, and other environmental, and social stimuli, to set circulating and local concentrations of TH, generally within narrow limits. This integration occurs in part at neurons that synthesize Thyrotropin-Releasing Hormone (TRH, pGlu-His-Pro-NH_2_) localized in the paraventricular nuclei (PVN) of the hypothalamus (11), nuclei bilaterally situated in the dorsal vicinity of the third ventricle (12). TRH neurons are localized in almost all parvocellular subdivisions of the PVN but only neurons present in the median and caudal regions of the PVN in rat (only median in mouse) are hypophysiotropic; their axons project to the external zone of the median eminence (ME) (13–15), the ventral part of the hypothalamus that connects it to the infundibulum. The median eminence forms a highly irrigated interface which serves both as a sensory and a secretory organ between the hypothalamus and the circulation (16).

Indirect yet complementary techniques have shown that TRH release from the median eminence is dynamic in multiple contexts. As studies on electrophysiological traces of TRH neuronal activity are scarce (17), many hypotheses on TRH neuron activity have been based on measurements of *Trh* mRNA levels (18, 19), and cFOS or phosphorylated cyclic-AMP response element binding protein (pCREB) induction in TRH neurons (10, 20, 21). Inferences about TRH release from ME have been made by measuring rapid changes in TRH content in ME (22). Information about the extracellular concentration of TRH came from the use of *in vivo* push-pull perfusion of the ME (23, 24) and surgical approaches to sample micro volumes of portal blood (25). Detailed descriptions of the inputs to TRH neurons, together with receptor localization and pharmacological tools (10) have led to a functional cartography of inputs onto TRH neurons, albeit their time resolution is poor (at best various min), and many unknowns remain.

Once released from hypophysiotropic nerve terminals into ME extracellular space, TRH enter fenestrated primary portal capillaries, which deliver it to the anterior pituitary *pars distalis*. Upon reaching the thyrotrope in the distal part of the anterior pituitary, TRH binds to TRH receptor 1 (TRH-R1), a G protein-coupled receptor (GPCR) expressed in pituitary thyrotropes (26). This interaction activates G_q/11_, increases intra-cytosolic calcium concentration and protein kinase C activity (27), and stimulates synthesis and release of Thyroid-Stimulating Hormone (TSH). TSH synthesis is regulated at levels of transcription and translation of α- and β-TSH subunits, their glycosylation and dimerization; bioactivity of released TSH depends on proper glycosylation (28, 29). The circulating concentration of TSH has been taken as a proxy for TRH secretion from ME, but the existence of multiple regulators of TSH secretion make firm conclusions difficult. Thus, Somatostatin (SRIF) neurons that originate in the periventricular nucleus of the hypothalamus (30) are other hypophysiotropic neurons involved in the central control of the HPT axis. The interaction of SRIF with its receptors, some of which are on thyrotropes (31), inhibits TSH secretion (32, 33). Although SRIF output can be modulated in ways consistent with a role in TSH control (34, 35), its function will not be further reviewed.

TSH reaches follicular cells of the thyroid gland and binds to the TSH receptor, a GPCR that stimulates the uptake of iodine and the activity of enzymes involved in the biosynthesis of 3,3′,5-triiodo-L-thyronine (T3) and thyroxine (T4); both are secreted (36). A major fraction of circulating TH is reversibly bound to carrier proteins in blood (37, 38), the small fraction of free TH can bind to membrane bound receptors (39) or enters cells through transporters (40). Multiple membrane transporters have the capacity to carry TH from the extracellular space into the cytosol, and vice versa. The most important is the Monocarboxylate Transporter 8 (MCT8, gene abbreviation: *Slc16a2*), which takes T4 and T3 from the extracellular space and the Organic Anion Transporter Polypeptide 1c1 (OATP1c1, gene abbreviation: *Slco1c1*), which has preference for T4 and reverse T3 (rT3) uptake (41).

T3 is the biologically active Iodo-Thyronine acting through nuclear TH receptors (TR); binding to α1-, β1-, or β2-TR controls transcription of multiple genes in almost all cell types (42). Local T3 concentrations depend mostly on its conversion from T4 by the tissue specific Deiodinases (D) type 1 and 2. D1 produces T3 by removing an iodine atom from the outer ring of T4 but can also remove it from the inner ring forming rT3, and deiodinates rT3 to T2. D2 catalyzes the transformation of T4 to T3. Finally, biologically inactive metabolites are produced by Deiodinase 3 (D3), which removes iodine from the T4 inner ring to produce rT3 or from T3 to produce 3, 3'-diiodothyronine, products with no binding affinity for TR (43). Extra- and intra-cellular carrier proteins, plasma membrane transporters and bio-transformations not only shape the local concentrations of TH, but also contribute to modulate their systemic effects (44).

Circulating TH generate negative feedback loops maintaining their serum concentration between set limits, although these limits can be changed according to metabolic challenges. A negative correlation exists between serum TH levels and *Trh* expression in the PVN (45–47). This negative correlation extends to TRH concentration in the PVN neurons (48, 49) and in portal vessels (25, 50, 51). The feedback depends on TH entering the brain through the MCT8 and OATP1c1 transporters (52–55), and on the interaction of β1-TR and β2-TR with T3 (28, 42), which are expressed in TRH neurons (56).

The basic HPT axis hierarchy is embedded in multiple regulatory circuits that adjust the local and global impact of TH according to physiological influences, or physio-pathological alterations (10, 11, 57, 58). A recently discovered level of HPT axis control relies on tanycytes, specialized ependymal cells present in sensory and secretory circumventricular organs (CVO) of the brain (16, 59), including the floor and the ventrolateral walls of the third ventricle (60–62). While astrocytes supply T3 to brain cells, tanycytes that border the dorso-, ventro-medial, and arcuate nuclei, as well as the median eminence, referred here as medio-basal hypothalamus (MBH) tanycytes, contribute to TH feedback on HPT axis, TH control of MBH circuits involved in energy homeostasis (10), as well as regulation of the amount of TRH entering the portal vessels (63, 64). We focus this review on the bidirectional pathways linking MBH tanycytes with TRH neurons activity and TRH entrance into portal vessels in mammals. We summarize knowledge about tanycytes and their phenotypic variation, demonstrate their critical involvement in TH feedback and adjustment of HPT axis activity according to energy related clues, introduce issues related to tanycyte programing of HPT axis and finally state some of the existing challenges in non-mammalian vertebrates.

## Multiple Types of Tanycytes Line the Ventral and Lateral Walls of the Third Ventricle

As ependymocytes, tanycytes have a small body lining some ventricle walls. In the MBH a long, basal process is directed to the hypothalamic parenchyma or blood vessels; MBH tanycytes have plenty of small and large protrusions full of endosomes directed to the third ventricle, which may have a secretory or transport function (60, 65). They express markers of caveolae- and/or clathrin- dependent endocytosis in their apical and basal domains, suggesting they can internalize molecules from the cerebrospinal fluid (CSF) and/or median eminence extracellular fluid (ECF) and transport some by transcytosis (66).

**Figure 1:**
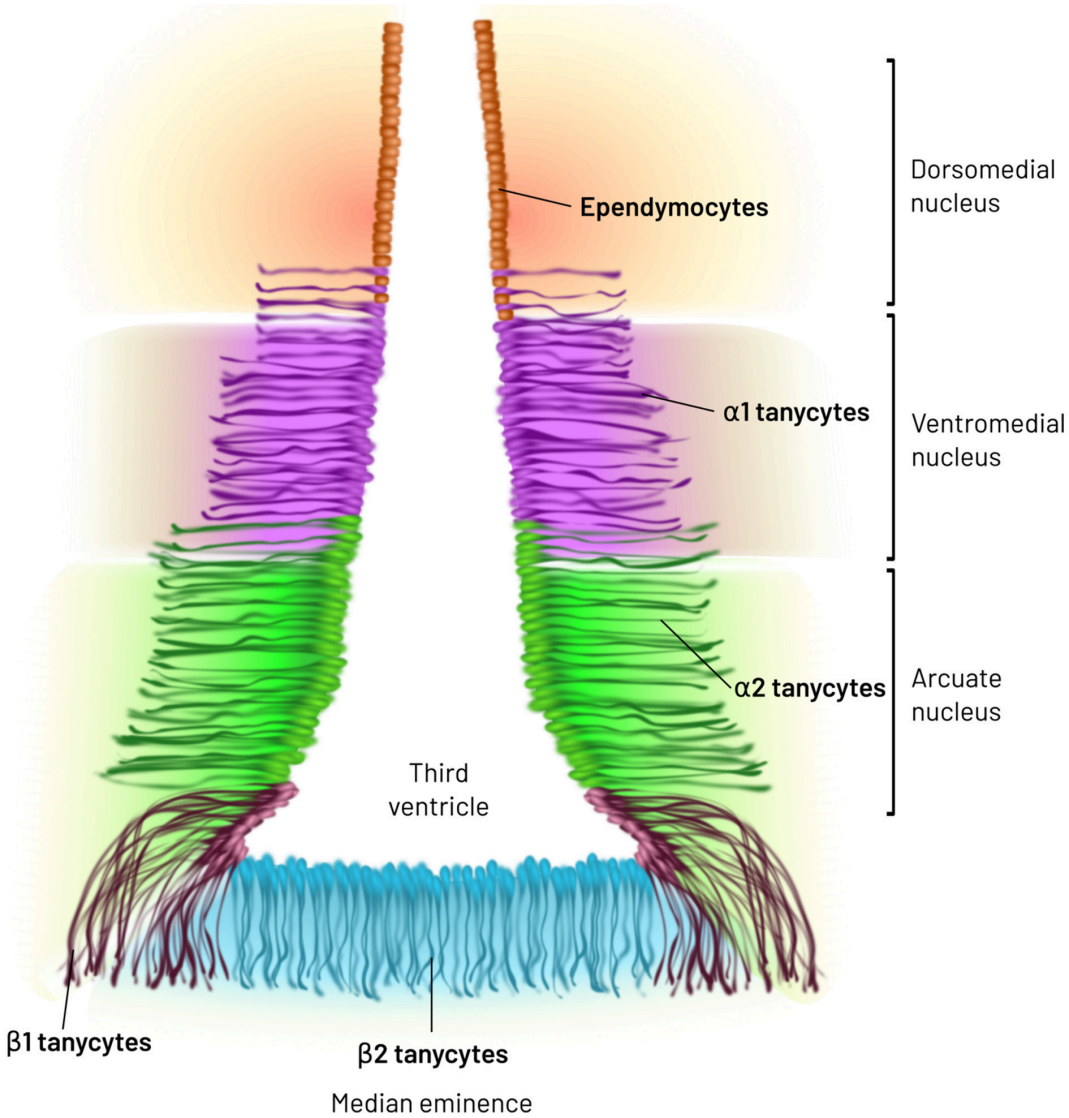
Major types of tanycytes in the medio basal hypothalamus. MBH tanycytes have been cataloged in 4 subtypes: α1-, α2-, β1-, and β2-tanycytes, according to location, expression of lineage and differentiation markers as well as based on mitochondria, tubular structures and secretory granules abundance. α1-Tanycytes extend their process to dorsomedial and ventromedial nuclei of the hypothalamus, while α2-tanycytes are restricted to the dorsomedial extent of the arcuate nucleus (ARC). β1- and β2-tanycytes reside in the ventral limits of the third ventricle.

MBH tanycytes have been cataloged in 4 subtypes: α1-, α2-, β1-, and β2-tanycytes, according to location, expression of lineage and differentiation markers (67), as well as based on mitochondria, tubular structures and secretory granules abundance (62). However, single-cell transcriptome and ultrastructural analyses suggest that each tanycyte subtype may be further subdivided (62, 68–70). α1-Tanycytes extend their process to dorsomedial and ventromedial nuclei (DMN and VMN) of the hypothalamus, while α2-tanycytes are restricted to the dorsomedial extent of the arcuate nucleus (ARC). At their basal pole, α-tanycytes contact and ensheathe laterally located blood brain barrier (BBB) vessels; cell bodies and initial segment of α-tanycytes additionally contact dendrites from ARC neurons (60, 62, 71). β1- and β2-tanycytes reside in the ventral limits of the third ventricle and their end-feet are proximal to fenestrated vessels of the ME; they form a barrier at their apical pole between the CSF and the periphery (62, 72) (Figure 1). In addition, β1-tanycytes delimit the borders of the adjacent ventromedial ARC and the ME. Along their processes they show interchained proteins, zonula occludens, and macula adherens, which join β1-tanycytes in bundles. This arrangement may contribute to a barrier that impedes the diffusion of molecules from the ME into the ARC in basal conditions (60, 71, 72), but a definitive functional evidence is lacking. Alternative mechanisms such as forces emanating from vessels cannot be discarded.

Despite stunning morphological and molecular similarities between radial glia and tanycytes, the latter are not radial glia but their descendants (60). MBH tanycytes are generated from hypothalamic progenitor cells in the last days of gestation and the first 2 weeks of life of the rat (60, 73). They express the intermediate filament proteins Nestin, Vimentin, and Glial Fibrillary Acidic Protein (74), the Dopamine- and cAMP-Regulated Phosphoprotein of 32 kDa (DARPP-32), a dopaminoceptive phosphoprotein (75), and proliferation and nuclear factors such as Antigen KI-67 and Sex Determining Region Y-Box 2 (74).

## Tanycytes and the Median Eminence

**Figure 2:**
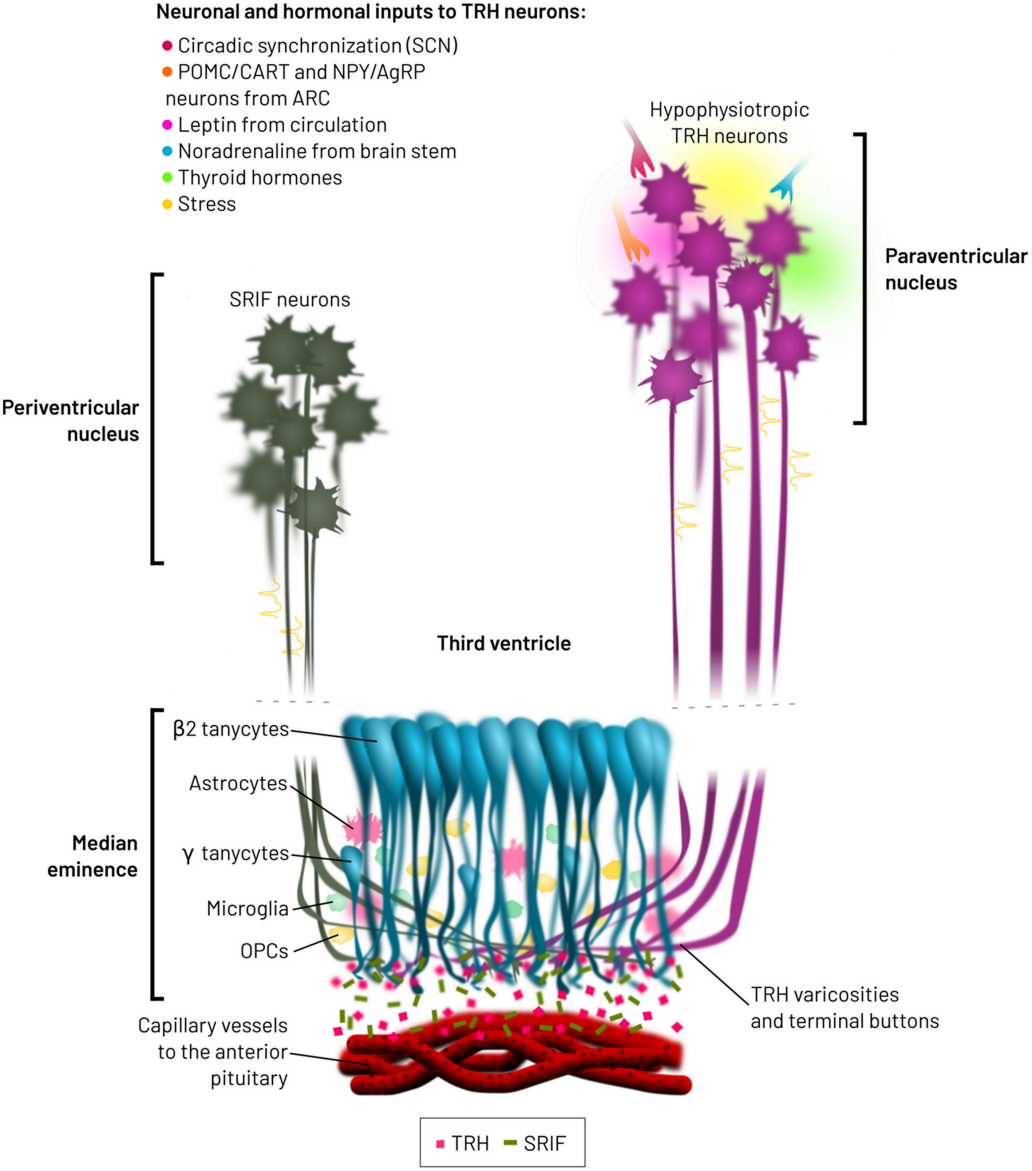
Spatial relationships between hypophysiotropic TRH and SRIF neurons, and β2-tanycytes. Hypophysiotropic TRH neurons from the PVN integrate multiple neuronal and hormonal inputs, which modify firing activity, TRH biosynthesis and receptor transduction pathways. SRIF neurons from the periventricular nucleus do also terminate in the medial median eminence. β2-tanycytes are located at the base of the third ventricle, from where they modify the bioavailability of TRH released from varicosities and terminal buttons, before entry into the lumen of the fenestrated capillaries. These capillaries transport TRH to the anterior pituitary, pars distalis, inducing the synthesis and release of TSH.

The ME is enriched with varicosities and terminal buttons from various hypophysiotropic neuron types, which release hypothalamic releasing factors into fenestrated capillaries directed to the pituitary. Furthermore, the ME harbors tanycytes, astrocytes, microglia, oligodendrocyte precursors and blood vessels (62, 76, 77) (Figure 2). Many interactions may occur between neuron varicosities and terminals and other cellular elements, either through juxtacrine or paracrine communication. Although frequency and pattern of action potentials generated in the hypophysiotropic neuron soma likely contribute to define the amount and pattern of peptides/transmitters released into the extracellular space of the median eminence external layer (78), modulation of stimulus-secretion coupling, action-potential independent secretion, post-secretory catabolism and barriers to diffusion or bulk flow may in addition alter the output of releasing factors into the portal vessels.

For Gonadotropin-Releasing Hormone (GnRH), strong evidences indicate various glial cell types and endothelial cells control its secretion in the lateral part of the median eminence. Astrocytes control GnRH secretion through paracrine signals, while GnRH secretion from neuron terminals proximal to the endothelial cells of the portal capillaries is potently regulated by nitric oxide produced by the endothelial cells. In addition, β1-tanycytes projecting into the ME have distal processes that terminate proximal to portal capillaries, with end-feet that can cover GnRH terminals and form a physical barrier reducing GnRH entry into portal vessels (62, 79–81).

**Figure 3:**
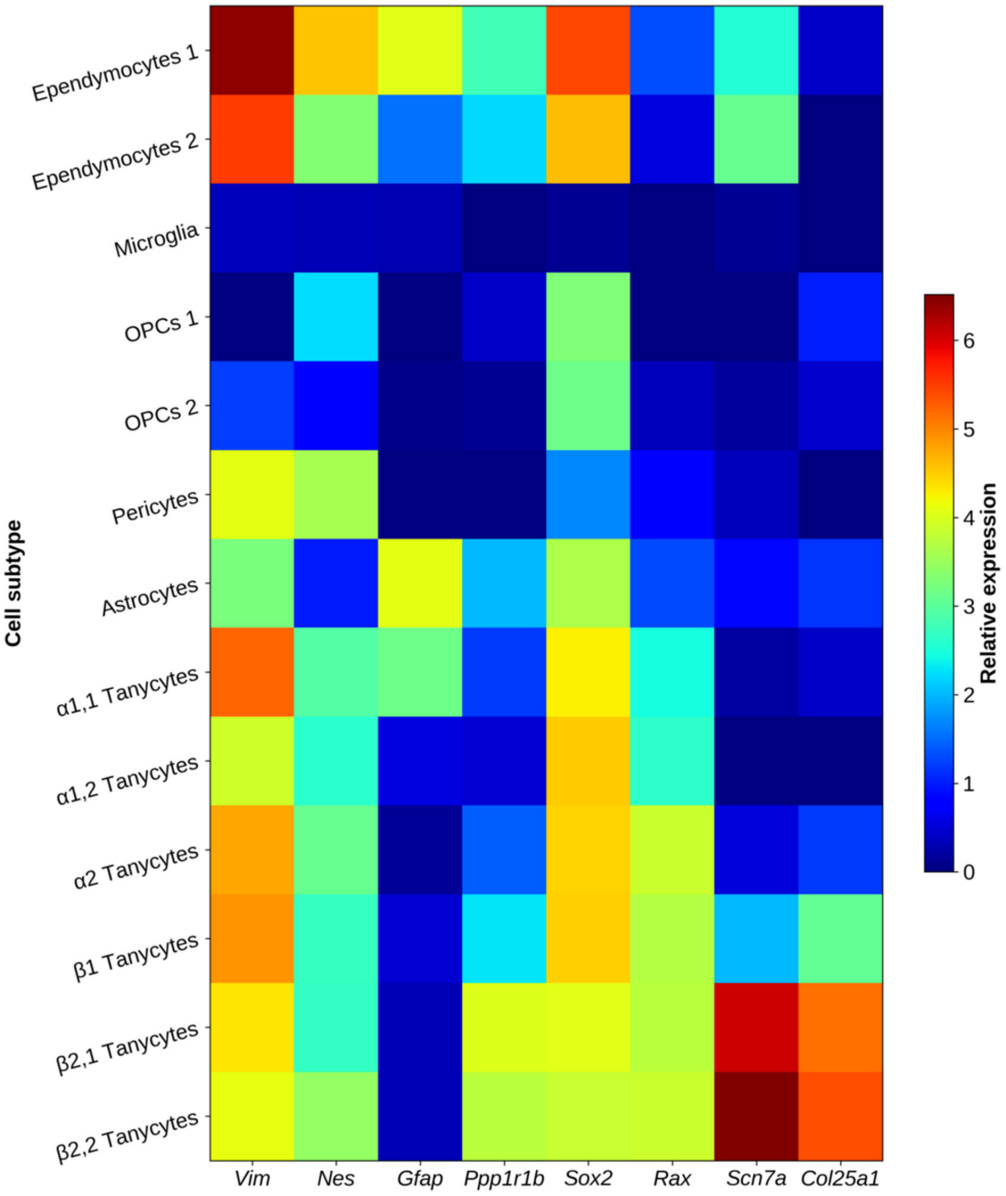
Heat map showing characteristic tanycyte markers in glial elements of the arcuate nucleus and the median eminence. MBH tanycyte clusters show an expression pattern enriched with structural genes (*Vim, Nes*), DARPP-32 (encoded by *Ppp1r1b* gene) and transcription factors *Sox2*, and *Rax*. β2-tanycytes are characterized by overexpression of *Scn7a* and *Col25a1*. Data represent the highest gene expression fold-change value in a cluster when compared to other neuronal and non-neuronal clusters of the arcuate nucleus/median eminence. Some cell types are subdivided in sub-clusters, since different expression patterns were found in that cluster. Data obtained from single-cell RNA-sequencing and transcriptome analysis in adult mice from Campbell et al. (69).

β2-tanycytes line the base of the third ventricle, with a distal process extended into the external zone of the medial part of the ME, where fenestrated portal vessels directed to the anterior pituitary are enriched. The apical process subdivides in a few branches in the external zone, and ultrastructural studies show these branches form numerous (100–200 per tanycyte) synaptoid contacts with peptidergic and aminergic vesicle-containing nerve buttons. Some β2-tanycytes have instead a basal process that projects into the *pars tuberalis* of the pituitary (62). Horseradish Peroxidase injected into the third ventricle diffuses freely into the hypothalamic parenchyma and has no access to the ME (60). β2-tanycytes are barriers between the ME and the third ventricle, as they express tight junction proteins like Zonula Occludens 1 and Occludin in their apical side, which form a honeycomb pattern (60, 72), impeding the free exchange of substances coming from the ME and the cerebrospinal fluid. Among tanycyte transcripts highly expressed in the β2-clusters, *Scn7a*, and *Col25a1* may be useful as specific markers of this subtype, since they are much less abundant in other tanycyte subtypes and glial cells (Figure 3). About 80% of terminal buttons arriving into the medial part of the external layer of the ME contain TRH (82), with an ample rostro caudal distribution terminating in the infundibular stalk (83). The antero-posterior and medio-lateral distributions of rat β2-tanycyte processes and TRH varicosities and terminals in the ME indicate a substantial spatial coincidence; in addition, synaptoid contacts between both cell types are observed (14, 63, 83, 84), suggesting that functional interactions occur. We will review the evidences that β2-tanycytes properties make them a critical cellular element of the HPT axis.

A putative fifth subtype of tanycytes, γ-tanycytes (85), previously identified as astrocytic or subependymal tanycytes (65, 86, 87) is localized in the ME. These cells resemble β2-tanycytes, including an abundant smooth and rough endoplasmic reticulum, yet their apical side does not contact the third ventricle, their processes are poor in microtubules and organelles (62), they express distinctive markers, including Propiomelanocortin (85), and contain lipid droplets in the perikaryon, which may be a source of the median eminence prostaglandins. It has been proposed that these tanycytes should be reclassified as pituicytes; their position, ultrastructure and contacts suggest they are relevant for neuroendocrine control (62).

## Tanycytes and Neurogenesis

Hypothalamic neurogenic niches have been observed in distinct populations of cells surrounding the third ventricle of the MBH, from ependymal cells to tanycytes. They act as progenitor cells that can differentiate into neurons or glia. New ARC, ventromedial and dorsomedial nuclei, and ME neurons may derive from tanycytes in postnatal animals. Thus, tanycytes may contribute to the programing/plasticity of adult hypothalamic circuits according to energy and/or nutritional signals [reviewed in Prevot et al. (81)]. Although unexplored, these events may impact on HPT axis regulation, as these MBH nuclei control hypophysiotropic TRH neurons.

## Tanycytes, the Hypothalamic Availability of Thyroid Hormones, and the Control of TRH Neurons Activity

More than 80% of adult brain T3 comes from deiodination of T4 (88–90). D2 is broadly distributed along multiple brain areas, expressed mostly in astrocytes, which capture T4 from blood vessels or CSF and deliver T3 to the neighboring neurons in the parenchyma. However, the MBH of euthyroid rats has a higher D2 activity than other brain areas (91). Cells expressing this deiodinase correspond to tanycytes, and to a lower extent to astrocytes (92, 93). In the ME, the transporters MCT8 and OATP1c1 are present in tanycyte processes (94, 95). Experiments with *Dio2* knockout mice contributed to elucidate the relative importance of the multiple cell sources of D2 for HPT axis regulation. Global *Dio2* knockout mice have elevated serum T4 and TSH concentrations, consistent with the necessity of this enzyme for negative feedback. However, *Trh* expression in hypophysiotropic neurons of the PVN remains unchanged. Mice with *Dio2* knockout specific for astrocytes have no detectable changes in thyroid axis hormones, demonstrating astrocyte D2 is not critical for hypophysiotropic function (96). Mice with a deletion of *Dio2* expression specific for the pituitary show high serum T4 and TSH concentrations, with unchanged D2 activity in the hypothalamus. However, *Trh* expression in PVN is decreased, indicating that remaining D2 tanycyte is critical for HPT axis negative feedback (96). In addition, immunohistochemical studies show a high expression of D3 in the median eminence; although mostly in GnRH axon terminals (97), *Dio*3-KO studies support the proposal this enzyme is necessary for normal HPT axis activity, in part through hypothalamic mechanisms (98–100).

**Figure 4:**
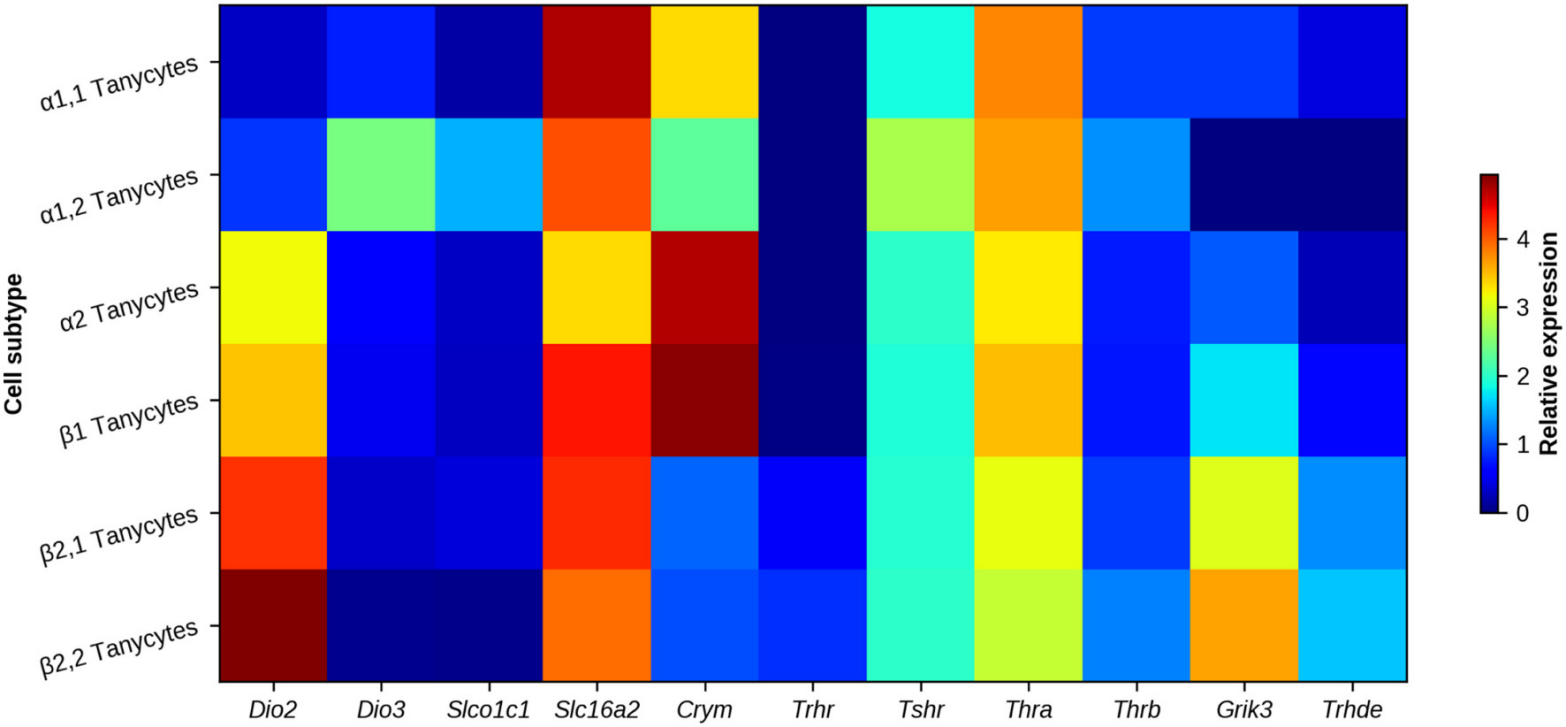
Heat map of genes associated with HPT axis regulation in MBH tanycyte subtypes. Regional differences can be observed between tanycyte subtypes. Deiodinases (*Dio2* and *Dio3*), TH transporters OATP1c1 and MCT8 (encoded by *Slco1c1* and *Slc16a2*, respectively), TH carrier *Crym*, receptors involved in HPT axis feedback/regulation (*Trhr, Tshr, Thra, Thrb*, and *Grik3*) and the TRH degrading ectoenzyme (*Thrde*) are mostly enriched in β2-tanycytes. Data represent the highest gene expression fold-change value of a tanycyte sub-cluster when compared to each non-neuronal cluster of the arcuate nucleus/median eminence. α1- and β2-tanycyte transcriptomes are each divided in two sub-clusters, corresponding to cell subtypes with a different gene profile. Data obtained with single cell transcriptome analysis in adult mice, from Campbell et al. (69).

TH bioavailability in the cytosol may also be under the control of μ-crystallin (CRYM), which binds with high affinity to T3 and T4 and functions similarly to serum TH transporters (101). The transfection of a plasmid expressing *Crym* in cells expressing MCT8 or MCT10 TH transporters increases the permanence of T3 in the cytosol (102, 103). Interestingly, *Crym* relative expression is higher in all subtypes of MBH tanycytes than in other cellular elements of the ARC/ME (69, 104), with a lower expression in β2-tanycytes. Differences in expression of *Crym* could adjust the time-course of T3 interaction with TR or the efflux of T3, and thus of target gene transcription. Figure 4 illustrates genes related with HPT axis regulation expressed in tanycyte subtypes.

The pathway that TH use to feedback on *Trh* expression in the PVN is still a puzzling matter. Although TH most probably reach the PVN cells through the BBB and *Dio2* mRNA is expressed in the PVN (105), possibly by astrocytes, early studies indicated that D2 is absent from PVN neurons (91, 92), and peripheral administration of a dose of T3 restituting its physiological levels is not enough to reduce *Trh* expression in the PVN in hypothyroid rats, unless larger doses are used (47). It was therefore put forward that after T4 entrance and T3 production by D2 in tanycytes, a route of T3 transport from tanycytes to the PVN was necessary for feedback regulation of TRH neurons. One proposal is that T3 could be transported from tanycytes to the third ventricle or parenchyma and bound to Transthyretin (106) diffuse, and/or move by bulk flow, to parvocellular PVN neuronal cell bodies. While ependymal cells proximal to the PVN have intercellular spaces that allow entry into parenchyma of practically all molecules from the ventricle, significant entry of T3 generated by tanycytes through this route seems unlikely. Tanycytes have 2 cilia (α-tanycytes) or 1 cilium (β-tanycytes), in contrast with the multiciliate ependymal cells in contact with the cerebrospinal fluid of the ventricles (107, 108). Tanycytes are poor contributors of cerebrospinal flow, as ciliary beat is involved with pulsatile motion of the cerebrospinal fluid from the ventricles (109). This observation suggests that in tanycyte-rich regions the CSF proximal to the ventricular walls is more static than in the upper ventricular wall, which is enriched with multiciliate ependymal cells; thus, molecules transported or generated by tanycytes may have a paracrine impact limited to proximate hypothalamic nuclei.

An alternate hypothesis takes into account that T3 exert feedback effects exclusively in the hypophysiotropic TRH neurons of the PVN located in middle and caudal zones, which send their axons to the ME, but not in nearby TRH neurons of the anterior PVN or lateral hypothalamus of the rat (15, 110). Since MCT8 and OATP1c1 are present in β2-tanycyte processes (95) and MCT8 is detected on axon varicosities of TRH neurons contacting tanycytes (97), these varicosities may be a preferential site of uptake for T3 by hypophysiotropic TRH neurons, followed by retrograde axonal transport to the PVN (10). Whether T3 is indeed retrogradely transported in TRH neurons requires further studies.

T3 generated from α-tanycytes may also interact with hypothalamic nuclei that regulate energy balance. D2 expressing α2-tanycytes may be in close relationship with AgRP neurons, and T3 increases the mitochondrial density and uncoupling activity in NPY/AgRP neurons of the ARC, actions related with their firing frequency. This may contribute to the feedback inhibition of TRH neuron activity, through the monosynaptic ARC-PVN pathway (5, 93) but also to TH-induced increase in food intake (111).

## Tanycytes, Thyroid Hormones Feedback and the Control of Median Eminence TRH Flux Into Portal Vessels

Another actor of HPT axis regulation expressed in tanycytes is pyroglutamyl peptidase II or TRH-degrading ectoenzyme (TRH-DE), a membrane-bound omega zinc-dependent metallopeptidase which catalyzes the hydrolysis of the pGlu-His bond of TRH in the extracellular space. Multiple evidences support the role of TRH-DE as the main regulator of TRH turnover in the extracellular space. TRH-DE specificity is narrow, hydrolysis being limited to pGlu-X-Y peptides (in which X is an uncharged residue and Y Pro, Ala, Trp, Pro-Gly, Pro-NH2, Pro-naphthylamide, or Pro-7-amino-4-methyl coumarin), being TRH the only biological substrate (112, 113). *Trhde* expression is mainly restricted to various brain regions, being particularly rich at ME site (114). Vimentin-expressing β2-tanycytes express *Trhde* in the cell body but possibly also along their basal process; moreover, *Trhde* expression is more intense in the external zone of the ME, where TRH neurons release their contents close to portal capillaries, than in other tanycytes domains (63). Single cell transcriptome analysis of MBH confirms *Trhde* is particularly enriched in β2-tanycytes (69) (Figure 4). The localization of *Trhde* mRNA to β2-tanycyte processes suggests that local translation of TRH-DE is a major source of TRH-DE activity in the intermediate and/or external layers of the median eminence. However, although TRH-DE activity is high in median eminence, coincident with *Trhde* mRNA levels (63), it should be noted that there are at least 2 isoforms of TRH-DE, one of them being a shorter, dominant-negative form; these isoforms are expressed in brain (115). Thus, clarification of the precise localization of TRH-DE activity in the β2-tanycyte domain awaits additional studies.

Functional evidence for TRH-DE relevance in regulating TSH secretion comes from experiments showing that inhibition of this enzyme enhances TRH recovery from incubation medium of median eminence explants (63), or in which the intra peritoneal (ip) injection of a brain-permeant inhibitor of TRH-DE (116) enhances serum TSH concentration in response to a cold stress or ip TRH (63). However, since the ip administration of the brain-permeant TRH-DE inhibitor may have altered TRH-DE activity in various tissues, definitive evidence for the importance of TRH-DE activity in tanycytes *in vivo* is lacking. For example, a soluble TRH-DE isoform generated in liver, termed Thyroliberinase (117), circulates in serum and may contribute to regulation of TSH secretion (118, 119). A link between TRH-DE and metabolism was first noted when it was shown that the activity of Thyroliberinase is positively correlated with body weight in man (120). More recently, single nucleotide polymorphisms of *Trhde* have been associated with differences in body weight and chest girth in sheep (121), but the phenotypic relevance of tanycyte *Trhde* is unknown.

TRH-DE activity is highly sensitive to TH levels in the median eminence, as previously demonstrated for pituitary and serum TRH-DE (122, 123). An ip injection of T4 to adult euthyroid rats enhances the expression of *Trhde* in tanycytes and of TRH-DE activity in the median eminence (63). Deiodination of T4 from D2 is necessary to change *Trhde* expression in response to short-term exposition (hours) to TH: in D2 knockout mice *Trhde* is upregulated by T3 administration but not by T4 (124). Changes in *Trh* mRNA levels in response to T3 are not as rapid as those observed for ME *Trhde* expression, suggesting that the regulation of *Trhde* expression in the median eminence by feedback may be critical for short term adjustment of TRH output (124). TH effects on *Trhde* expression are likely direct on tanycytes since they express *Thra* [(69), Figure 4].

Since recent evidence suggests that control of β2-tanycyte end-feet morphology impacts serum TSH concentration (see section “Interactions between β2-tanycytes and hypophysiotropic TRH neurons directly control the output of TRH into portal vessels”), it seems appropriate to test whether morphological changes in the end feet of tanycytes occur in response to TH, and contribute to the feedback control of the HPT axis in mammals.

Finally, because TH control proliferation and differentiation of progenitor cells to a neuronal phenotype in adult rodents (125–127), and tanycytes have the molecular machinery needed to integrate TH signaling, testing whether fluctuations in local TH levels in the MBH may control the neuron precursor potential of tanycytes is warranted (128). Apart from negative feedback, other energy related cues regulating HPT axis activity implicate tanycytes.

## Cold Exposure, HPT Axis, and Tanycytes

In response to cold exposure, catecholaminergic pathways from the brainstem activate a large subpopulation of hypophysiotropic TRH neurons leading to enhanced secretion of TRH and TSH secretion, and TH synthesis (11, 18, 19, 51, 63, 129–131), contributing to facultative thermogenesis, a critical event for body thermoregulation. Although responses of the HPT axis to cold have been attributed to regulation of TRH neurons activity, additional events occurring in the median eminence level have also been detected, including evidence that during a cold stress an interaction between NA and TRH terminals (132) plays a permissive role for TRH secretion (23, 133). Tanycytes may also regulate the dynamics of TRH entry into portal capillaries during a cold stress since the ip administration of a TRH-DE inhibitor enhances serum TSH concentration induced by cold exposure (63).

## Negative Energy Balance, HPT Axis, and Tanycytes

The ARC exerts a well-known and critical influence on homeostatic mechanisms of energy intake and expenditure. The activity of ARC neurons expressing POMC/CART or NPY/AgRP/GABA is regulated in opposite direction according to nutritional status and energy balance, in direct response to signals such as leptin, ghrelin, insulin, and glucose. ARC neurons provide direct inputs onto hypophysiotropic TRH neurons, and their messengers directly up- (αMSH, CART) or down-regulate (NPY, AgRP) TRH neurons activity (10, 17, 134).

### Fasting, Food Restriction, and Voluntary Exercise

Fasting promotes a profound down regulation of HPT axis activity, sparing energy use. This is driven by a reduction of PVN *Trh* mRNA levels and TRH concentration in portal vessels, leading to decreases in serum TSH and TH concentrations (10). In this model, the reduction of TRH neurons activity has been attributed in part to the effect of a decrease of circulating leptin concentration (135), mediated by inhibition of the POMC/CART neurons of the ARC and stimulation of the NPY/AgRP/GABA neurons (10, 136, 137), and also by reduction of direct leptin stimulation of TRH neurons (138, 139). On the other hand, high levels of circulating ghrelin in fasted animals may also indirectly inhibit the activity of TRH neurons (140, 141), although this is not settled.

Leptin effects on the central arm of the HPT axis depend on access to the hypothalamic parenchyma. Although the ventromedial ARC has vessels whose permeability is regulated by energy status (142), making part of this nucleus sensitive to circulating peptides during fasting, peptide hormones do not generally pass the BBB, suggesting additional mechanisms of transport must operate (143). Thus, leptin enters the brain bypassing the BBB across the choroid plexus (144), and through median eminence tanycytes. Transport of leptin through tanycytes into the CSF depends on Extracellular Regulated Kinase induction in tanycytes (145). Tanycytes may also transport ghrelin from the median eminence into the CSF (146, 147). Peptide hormones can easily diffuse from the ventral part of the third ventricle, into the adjacent dorsomedial ARC, and possibly via the parenchyma to the PVN. Thus, the function of critical regulators of HPT axis activity likely depends, at least in part, on the transport function of tanycytes.

During fasting, *Dio2* mRNA expression and activity is upregulated in tanycytes (148), in parallel with a local increase in T3 concentration (5), albeit this increase is transitory, it coincides with the lowest levels of *Trh* expression in the PVN (119), consistent with a role of tanycyte D2 in the local regulation of the HPT axis. Possibly because of this induction, an increase of ME TRH-DE activity is detected in response to a prolonged (72 h) fast in male rats; that may strengthen the reduction of HPT axis activity (119).

Therefore, tanycyte D2, and TRH-DE coordinated regulation during prolonged fasting likely contributes to maintain inhibition of HPT axis activity (119). The increase in D2 activity raises the local levels of T3 which feedbacks on TRH synthesis, while up regulation of the expression of *Trhde* and its activity in the tanycyte may reduce TRH access into the portal blood. Fasting-induced increase in D2 activity could also regulate the activity of ARC neurons, thus indirectly controlling the HPT axis (5). However, since *Dio2* expression in the PVN is upregulated by fasting (105), other hypothalamic sources of T3 may also contribute to regulate the activity of TRH neurons when energy balance is negative.

Food reduction or restriction can also reduce the activity of the HPT axis (149–152). Contrary to fasting, a strong food restriction (gradually from 35 to 75% for 7 days) decreases *Trhde* expression, but not TRH-DE activity, although D2 expression and activity are increased in the MBH (152).

Compared to sedentary animals, 2 weeks of voluntary exercise in male rats diminish food intake by 18% and markers of the central activity of the HPT axis, increase the activity of D2 in MBH but have no effect on *Trhde* expression in the median eminence (153). Thus, as occurs during food restriction, sustained but limited negative energy balance does not increase the expression of *Trhde* in the ME, implying changes in *Trhde* expression in the ME may depend on intensity and/or duration of negative energy balance.

### Non-thyroidal Illness Syndrome

Pathological conditions, as chronic infection or cachexia produce the non-thyroidal illness syndrome (NTIS) generally characterized by normal basal TSH concentration, and low thyroid hormone serum concentrations (154). Experimental evidences indicate central and peripheral changes. The injection of bacterial Lipopolysaccharide in animals, which mimics a bacterial infection, suppresses hypophysiotropic *Trh* expression and serum TH concentrations (10, 84, 148, 155). Unlike fasting, endotoxin injection has a pronounced positive effect on *Dio2* expression in the α-tanycytes (84, 156), the peak in *Dio2* expression coincides with the maximum decrease of *Trh* mRNA level in the PVN, suggesting that, apart from the β-tanycytes, α-tanycyte T3 production is also critical for HPT axis regulation (84, 148, 155).

## Interactions Between β2-Tanycytes and Hypophysiotropic TRH Neurons and the Flux of TRH Into Portal Vessels

The previous sections have shown that tanycytes determine local concentrations of TH, sense and transport energy related cues, and may control TRH turnover in the extracellular space of the ME, all of which influence directly or indirectly TRH neurons activity and output into portal vessels. New evidences suggest TRH neurons control tanycyte properties defining the output of TRH into the portal capillaries, through mechanisms which operate in the external layer at post-secretory levels. While a hybridization signal for TRH receptors was not initially detected in the median eminence (26), more recent evidence indicates β2-tanycytes do express low levels of *Trhr* [(64, 69), Figure 4]. Activation of TRH-R1 in β2-tanycytes induces Ca^++^ entry and an increase of TRH-DE activity in the median eminence. This may enhance TRH hydrolysis before entry into the portal vessels, a decrease in bioavailability which may limit desensitization and/or downregulation of TRH-R1 in the thyrotropes, and/or contribute to transient pulses of TRH. These experiments also showed that TRH binding to TRH-R1 promotes the extension of tanycyte basal processes between TRH terminals and portal vessels, which may also reduce the flux of TRH into the portal vessels. Therefore, TRH seems to have the capacity to modulate its own entry into portal vessels by two complementary mechanisms: modulation of TRH-DE activity and end-feet contacts of β2-tanycytes with portal capillaries (64). These evidences were obtained in part in models in which Gα_q/11_ proteins were made inactive; however, the functional demonstration that TRH-DE activity and/or end-feet contacts of β2-tanycytes with portal capillaries are critical is still lacking.

Apart from TRH, hypophysiotropic TRH neurons use glutamate as a transmitter (157). The role of this pool of glutamate is still under investigation, but it is interesting to note that mRNA coding for 2 ionotropic glutamate receptors, including the Glutamate Receptor Ionotropic Kainate Type Subunit 3, are expressed by tanycytes [(69, 158, 159), Figure 4], and that glutamate regulates TRH-DE activity in the hippocampus (160), making it tempting to speculate glutamate regulates TRH-DE activity in the ME.

## Programming of HPT Axis and Tanycyte *Trhde* Expression

In the rat, the development of median eminence tanycytes, which are first detected before birth (87) occurs in parallel with multiple aspects of HPT axis ontogeny. Since tanycytes control the local (hypothalamic) bioavailability of TH, the postnatal development of tanycytes is probably critical for HPT feedback development, as has been suggested in chicken (161).

Multiple determinants, which include nutrition, stress and toxics exposure, during pre- or post-natal development can program adult HPT axis function (162, 163). Some impacts have been linked to hypophysiotropic TRH neurons and tanycytes, although the mechanistic insights are still limited (163). One of the best understood models of post-natal stress is repeated maternal separation (MS) during lactation. MS causes multiple long-term endocrine perturbations (164), including the functional state of the HPT axis in adult rats in a sex related manner. Pups separated from their mother for 3 h daily during lactation have altered HPT axis activity. As adults, male rats have decreased TSH and T3 serum concentrations and a higher expression of *Trhde* in tanycytes of ME, compared to undisturbed pups. MS males do not respond to fasting as expected: *Trhde* expression is not enhanced and HPT axis activity inhibition is blunted. These changes are not detected in MS females who have higher (compared to undisturbed animals) fat mass and *Trh* expression in PVN but normal serum concentrations of TH and no changes in their reaction to fasting (165). The higher susceptibility to MS of males compared to females has been observed in other paradigms (166), but it is interesting that the more intense change is in *Trhde* expression and is long-lasting (165). The sex- specific programing of *Trhde* expression together with TSH and T3 serum concentrations in this MS paradigm reinforces the hypothesis that alterations in tanycyte properties can have short- and long-term consequences on thyroid status. Understanding the mechanisms programing tanycyte functions, including *Trhde* expression, in response to stressors, nutrition and toxic substances is warranted.

## Tanycytes and HPT Axis in Non-Mammalian Vertebrates

The evolutionary origin of the TRH neuron-tanycyte interaction is poorly understood. Except for teleosts, hypothalami of vertebrates have a median eminence whose external layer connects hypothalamus and pituitary via a portal system. As in mammals, in most non-mammalian vertebrates, the external layer also contains end-feet of glial-like cells, which cell body is localized in the floor of the third ventricle (167). These glial-like cells express vimentin and GFAP (therefore denominated tanycytes) and contact portal vessels in most vertebrates (168–170). Moreover, in non-mammalian vertebrates, tanycytes of the median eminence have an anatomical position akin to that seen in mammals, although their molecular signatures are unknown. Since most non-mammalian vertebrates possess a median eminence with tanycytes analogous to β2-tanycytes, they are probably fundamental to create an appropriate physical, molecular and anatomical link between brain and pituitary, and regulate the HPT axis.

Although *Trh*, and *Trhr* genes are detected in most non-mammalian vertebrates, their functional roles are not always related to the regulation of TSH secretion (171), and therefore to the control of TH secretion. Thus, for example, the thyroid status does not regulate TRH synthesis in the hypothalamus of the fish brain (172, 173), in a site homologous to the mammalian PVN. On the contrary, Corticotropin Releasing Hormone (CRH) is a major Thyrotropin-Releasing Factor (TRF) in non-mammalian vertebrates (174). Among the many unknowns about non-mammalian vertebrates tanycytes and HPT axis, we can pinpoint the following: could the local production of T3 regulate hypothalamic CRH synthesis, as it does for TRH in mammals? Are morphological changes of tanycytes regulating TRF availability? Is an hydrolase analogous to TRH-DE operating for another TRF?

## Photoperiod and the Control of Tanycyte-Derived T3

In avian and mammalian species sensible to photoperiod, photoperiod effects on neuroendocrine axes critically depend on tanycytes. Melatonin, which transduces photoperiod, interacts with the melatonin 1 receptor expressed in secretory *pars tuberalis* specific thyrotropes, a cell phenotype different from the *pars distalis* thyrotropes, and induces rhythmic TSH secretion from the *pars tuberalis* linked to the regulation of seasonal breeding (175, 176). Exposure to long days enhances TSH secretion from the *pars tuberalis* (177–179), followed by a later stimulation of *Dio2* and a decrease of *Dio3* expression in tanycytes (180, 181). Although all tanycyte subtypes express *Tshr* (Figure 4), TSH-induced expression of *Dio2* occurs mostly in α1-, α2-, and β1-tanycytes (177–179). As a result, MBH T3 levels are raised in long day seasons (180). This in turn influences the morphology of tanycytes end-feet in the lateral median eminence, increasing GnRH terminals access to the basal lamina and release into the portal circulation (182, 183). *Pars tuberalis* thyrotrope interaction with MBH tanycytes and reproductive axis consequences have been reviewed recently (184).

Although TSH-induced expression of *Dio2* during seasonal cycles is limited to α- and β1-subtypes, it may still influence *Trh* transcription in the PVN if β2-tanycytes are not critical for T3 effects on the HPT axis (see for example sub section “*Non-thyroidal illness syndrome”*). Nevertheless, photoperiod has no effect on *Trh* expression in Siberian hamster or F344 rats (185, 186). Furthermore, TSH production from the *pars tuberalis* is not affected by thyroid hormones nor TRH (175) and even if *pars tuberalis*-derived TSH can bind to TSH receptors, it is not active in the thyroid gland (187). Thus, TSH released from the *pars tuberalis* has only a local action in the MBH, and there is no evidence that it regulates the HPT axis activity according to photoperiod. The lack of coupling between TSH receptor activation and *Dio2* expression in β2-tanycytes may result in uncoupling of the HPT axis from photoperiod information (184).

## Conclusions

**Figure 5:**
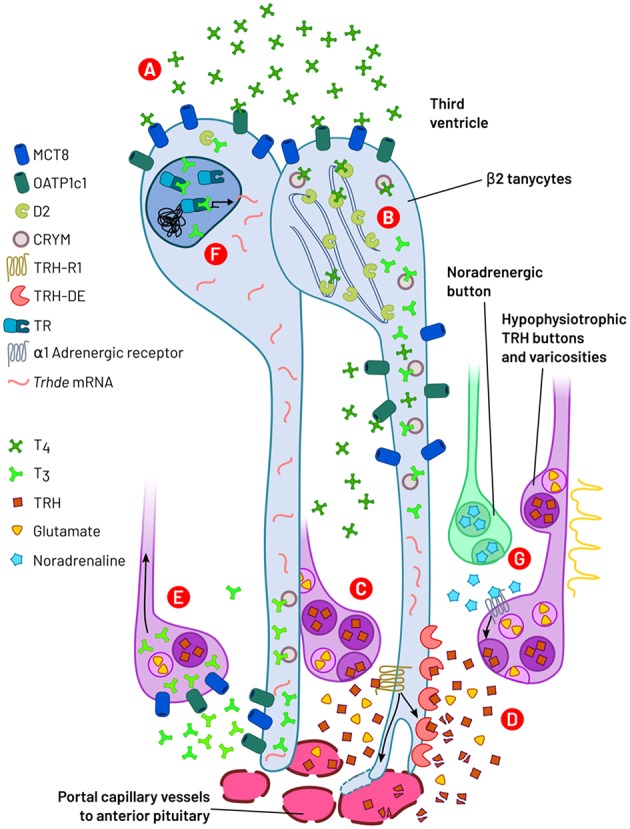
A summary of events operated through β2-tanycytes and axon terminals regulating TRH output from hypophysiotropic neurons. (A) Third ventricle/extracellular medium T4 is internalized into β2-tanycytes by MCT8 and OATP1c1 transporters. (B) In β2-tanycytes, THs are bound to CRYM and T4 is bio-transformed to T3 by deiodinase 2 (D2), an enzyme localized in the endoplasmic reticulum. (C) Action potential arrival or local signals promote TRH and glutamate release into the median eminence extracellular compartment by exocytosis. TRH binds to TRH-R1 receptors localized on β2-tanycyte end-feet, increasing TRH-DE activity and tanycyte end-feet expansion. (D) Membrane bound TRH-DE hydrolyses TRH. Both actions (C,D) control TRH bioavailability and output to the anterior pituitary. (E) T3 is transported out of tanycytes into the extracellular space and may be captured through MCT8 and OATP1c1 into TRH terminal buttons/varicosities; retrograde transport of T3 may in turn inhibit *Trh* synthesis at somatic level. (F) Binding of T3 to tanycyte nuclear TR may increase the expression of *Trhde*. (G) Noradrenaline stimulates TRH secretion through α1-adrenergic receptors, possibly localized on TRH varicosities/terminal buttons.

In the external layer of the median eminence, various imbricated inter- and intra-cellular processes may coordinate the flux of TRH into the portal capillaries, down-stream of the enhancement of TRH release by action potentials arrival. Tanycytes are critical cells that link HPT axis activity to physiological status through molecular and cellular and adaptations. Thus, although TH negatively regulate hypophysiotropic TRH neurons activity, this role depends on biotransformation of T4 in tanycytes. Furthermore, the post-secretory availability of TRH may also be TH dependent, regulated by tanycyte TRH-DE activity and possibly the physical barrier that the end-feet make near the portal vessels. These advances suggest that tanycytes, in particular β2-tanycytes, are critical for feedback control of the HPT axis (Figure 5). An interesting question remaining to be solved is the relative importance for TRH output in mammals of the barrier function of the end-feet and of TRH-DE activity.

Another remarkable aspect is that TRH, and possibly glutamate, released from TRH terminals, regulates TRH accessibility to portal vessels through a dynamic and reciprocal interaction with tanycytes. This interaction rapidly regulates the activity of the TRH-DE and the end-feet contacts with median eminence capillaries. On the other hand, tanycytes may also feedback on TRH secretion, interactions that together may contribute to generate cycles of TRH release, and/or may rapidly regulate it in response to physiological stimuli, such as during cold exposure. Median eminence tanycytes are thus an additional critical level of control of the HPT axis, sensitive to energy balance clues, and impacting on TRH output. The efficiency of this control point may be programmed by developmental challenges.

Other aspects of HPT axis control at median eminence level still requiring investigation are putative interactions of SRIF terminals and tanycytes, since there is an ample rostro caudal distribution of SRIF varicosities that terminates in the infundibular stalk (30). These and other local mechanisms may also have a significant effect on the control of the thyroid axis.

Finally, although knowledge about the relation of tanycytes and HPT axis function is still limited, it is tempting to think that clinical applications may be considered in the future, since the ME compartment is outside the BBB. Sex dimorphism should be investigated, because of its physiological and clinical relevance.

## Author Contributions

AR-R and J-LC conceived and wrote the manuscript. IL, ES-J, RU, LJ-H, and PJ-B wrote selected portions and edited the manuscript. All authors read and approved the final manuscript. Figures were made by AR-R with open source software, including Inkscape (www.inkscape.org) and Matplotlib (188).

### Conflict of Interest Statement

The authors declare that the research was conducted in the absence of any commercial or financial relationships that could be construed as a potential conflict of interest.
